# The action of enhancing weak light capture via phototropic growth and chloroplast movement in plants

**DOI:** 10.1007/s44154-022-00066-x

**Published:** 2022-12-01

**Authors:** Guang-yuan Xin, Lu-ping Li, Peng-tao Wang, Xin-yue Li, Yuan-ji Han, Xiang Zhao

**Affiliations:** State Key Laboratory of Crop Stress Adaptation and Improvement, School of Life Sciences, Henan University, Kaifeng, China

**Keywords:** Blue light, Chloroplast movement, Phototropic growth, Phototropin1, Phototropin2

## Abstract

To cope with fluctuating light conditions, terrestrial plants have evolved precise regulation mechanisms to help optimize light capture and increase photosynthetic efficiency. Upon blue light-triggered autophosphorylation, activated phototropin (PHOT1 and PHOT2) photoreceptors function solely or redundantly to regulate diverse responses, including phototropism, chloroplast movement, stomatal opening, and leaf positioning and flattening in plants. These responses enhance light capture under low-light conditions and avoid photodamage under high-light conditions. NON-PHOTOTROPIC HYPOCOTYL 3 (NPH3) and ROOT PHOTOTROPISM 2 (RPT2) are signal transducers that function in the PHOT1- and PHOT2-mediated response. NPH3 is required for phototropism, leaf expansion and positioning. RPT2 regulates chloroplast accumulation as well as NPH3-mediated responses. NRL PROTEIN FOR CHLOROPLAST MOVEMENT 1 (NCH1) was recently identified as a PHOT1-interacting protein that functions redundantly with RPT2 to mediate chloroplast accumulation. The PHYTOCHROME KINASE SUBSTRATE (PKS) proteins (PKS1, PKS2, and PKS4) interact with PHOT1 and NPH3 and mediate hypocotyl phototropic bending. This review summarizes advances in phototropic growth and chloroplast movement induced by light. We also focus on how crosstalk in signaling between phototropism and chloroplast movement enhances weak light capture, providing a basis for future studies aiming to delineate the mechanism of light-trapping plants to improve light-use efficiency.

## Introduction

Plants respond to environmental stimuli and use environmental signals to guide their growth and development. Light is a key environmental factor controlling plant growth and morphogenesis. Plants contain various photoreceptors that can sense the direction, quality, and intensity of incident light and make corresponding adjustments to adapt to the environment (Hohm et al., 2013). Plants are often exposed to light stress during natural growth. High-intensity light can cause organelle damage, whereas weak light cannot satisfy the requirements for photosynthesis. Similar issues are observed during agriculture: extreme light conditions can induce light stress in crops. Over-dense planting of crops can lead to insufficient light, thereby hindering crop bioaccumulation. High-intensity continuous irradiation damages organelles, accelerates water evaporation, and leads to wilt. Therefore, studies are needed to evaluate photoresponse patterns in plants. Phototropins (PHOT1 and PHOT2) act as photoreceptors in plants and respond to environmental conditions by regulating phototropism, chloroplast movement, stomatal opening and leaf extension (Christie, 2007).

Phototropism allows plants to orient their photosynthetic organs toward the light and this behavior also occurs in mosses, ferns, and angiosperms (Takemiya et al. 2005; Suetsugu and Wada, 2007b; Goyal et al. 2013; Hohm et al. 2013; Briggs, 2014). Plants can perceive a broad spectrum of light, from ultraviolet to far-red; however, only ultraviolet-B and blue light can induce phototropism (Liscum et al., 2014; Fankhauser and Christie, 2015). Under blue light, phototropism of the Arabidopsis hypocotyl is regulated mainly by phototropins in the inner cytoplasmic membrane. Blue light can induce the phosphorylation of PHOT1 and promote the detachment of NPH3 from cell membrane, and RPT2 is induced by blue light to interact with NPH3 and PHOT1 to promote phototropism (Sakai et al., 2001; Morita and Tasaka, 2004; Stone et al., 2005). Phytochrome A (PHYA) and cryptochromes (CRYs) can also sense blue light signals. These proteins are localized in the nucleus and indirectly affect the phototropism of hypocotyls by regulating the transcription of related genes (Kang et al., 2008; Nagashima et al., 2008b; Goyal et al., 2013; Liscum et al., 2014).

Chloroplast migration occurs in all plant species ranging from algae to terrestrial plants (Kataoka, 2015). Chloroplasts of various plants are found in different locations in the cells under different light conditions (Banas et al., 2012; Wada, 2013). The locations of chloroplasts dynamically vary in response to the position and intensity of incident light through low-intensity light-induced accumulation responses and high-intensity light-induced avoidance responses. Phototropins, as blue light receptors, can also mediate chloroplast movement (Kagawa et al., 2001; Kagawa and Wada, 2002). In most plants, blue light is the most effective wavelength for inducing two reactions of chloroplast motility, including in angiosperms (e.g., Arabidopsis, spinach, tobacco) (Davis and Hangarter, 2012) and cryptogamic plants (e.g., ferns, mosses, lichens, algae) (Suetsugu and Wada, 2007b). In contrast, red light induces chloroplast movement in several cryptogam plants, including the alga* Mougeotia scalaris*, the fern* Adiantum capillus-veneris*, and the moss* Physcomitrella patens* (Schönbohm, 1980; Suetsugu and Wada, 2007b; Wada, 2008). Research on *Arabidopsis thaliana* has revealed the signaling mechanism of phototropin-mediated plant movement. However, the role of phototropins in crops has not been widely examined. This review focuses on the molecular mechanisms of phototropin-mediated phototropism and chloroplast movement, and progress in research on phototropin-mediated motility in crops, and how crosstalk improves light efficiency in plant.

## Phototropins as light receptors activated by blue light

**Fig. 1:**
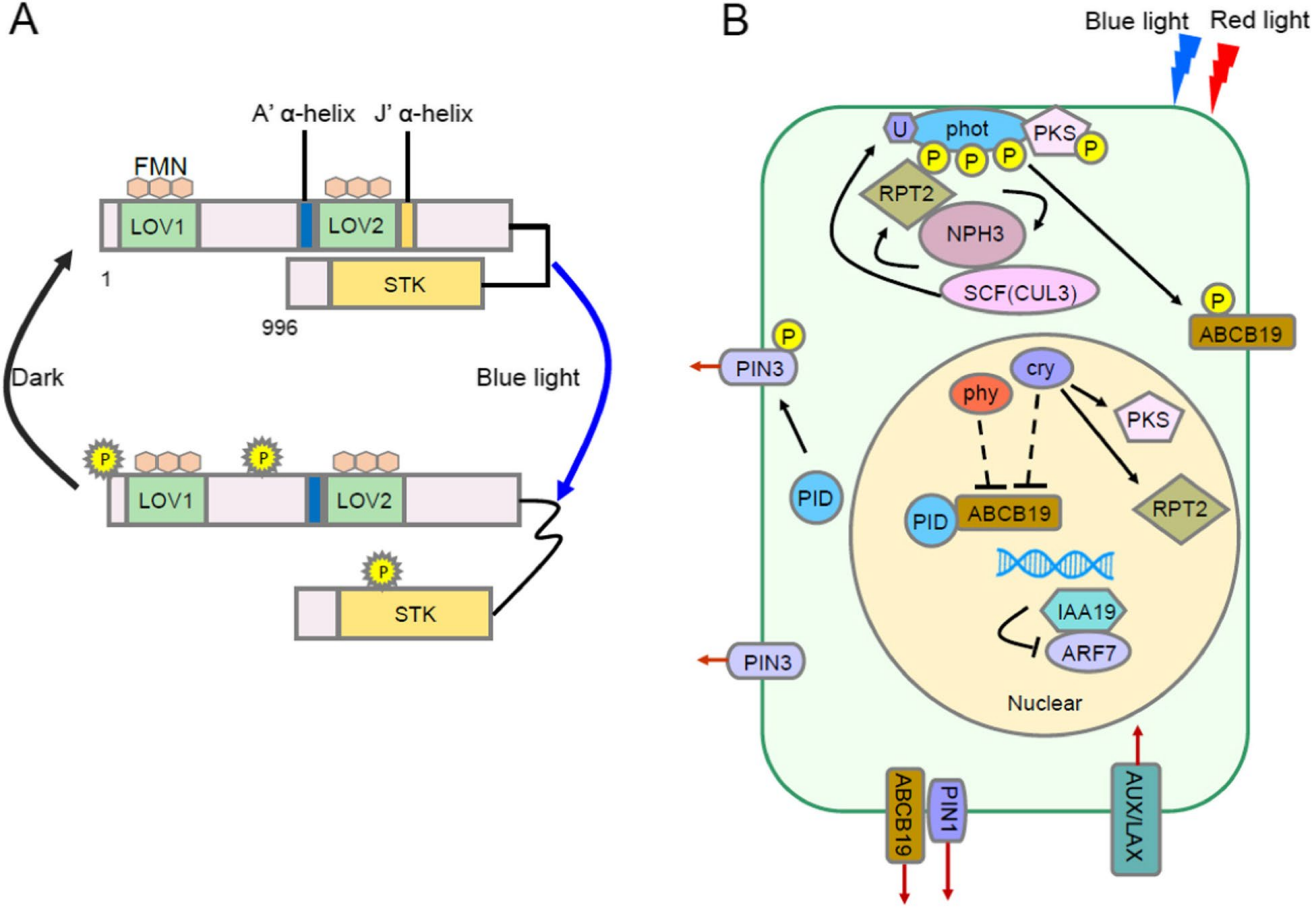
Schematic diagram of the reversible activation of PHOT1 under blue light and the signal transduction of light-induced phototropism. **A** phot1 and phot2 have similar structures. they have two LOV domains at the N-terminus and one STK kinase domain at the C-terminus, and non-covalently bound to the FMN chromophore. In the dark, LOV2 interacts and inhibits the activity of STK. Blue light can induce LOV and FMN from non-covalent to covalent binding, resulting in a conformational change, STK activation, and phosphorylation of PHOT1. phot2 is not shown in this figure and has similar protein structures with phot1. **B** Blue light can induce hypocotyl phototropism in Arabidopsis seedlings, which is caused by asymmetric distribution of auxin in the hypocotyl. In this process, blue light can activate PHOT and initiate a series of molecular events regulating phototropism. In the dark, both phot1 and NPH3 localize to the cell membrane and interact. Blue light can dephosphorylate and internalize aggregation of NPH3. Prolonged blue light irradiation or dark treatment can phosphorylate and relocate NPH3 to the cell membrane. Intense blue light can induce the expression of RPT2 and PKS4. RPT2, PKS4 can form complexes with PHOT1 and NPH3. The E3 ubiquitin ligase compound SCF formed by CUL3 can interact with NPH3 and participate in the ubiquitination of PHOT1 induced by blue light. Blue light activates PHOT1 and induces phosphorylation of ABCB19 and PKS4, inhibiting its activity, thereby enhancing phototropism. ABCB19 can interact with PIN1 and regulate its membrane localization. PID can also modulate the phosphorylation status of PIN3 and affect its localization. As an important auxin response factor, ARF7 can regulate auxin distribution, and the transcriptional repressor IAA19/MSG2 can bind to ARF7 and inhibit its activity. On the other hand, phytochromes and cryptochromes can mediate the transcription of related genes under blue and red light, respectively, thereby indirectly affecting phototropism. Red arrows indicate the direction of auxin transport, black arrows indicate molecular action. P: phosphorylation U: ubiquitination

Phototropins are serine or threonine protein kinases activated by light and belong to the cAMP-dependent protein kinase, cGMP-dependent protein kinase G, and phospholipid-dependent protein kinase C (AGC) kinase families (Bogre et al., 2003). Phototropins are the primary photoreceptors for blue light-mediated phototropism (Liscum et al., 2014). Model plant Arabidopsis contain two homologues of phototropin, phot1 and phot2. Phototropins are composed of approximately 1000 amino acids and two flavin mononucleotides (FMN) domain, and their *N*-terminus contains two repeating conserved sequences, LOV1 and LOV2. The *C*-terminus of phototropin is a serine/threonine kinase domain (STK) that interconnects with LOV2 (Christie, 2007; Okajima, 2016). The native phototropins is reportedly a dimer, with the LOV1 domain responsible for dimerization (Salomon et al., 2004). The LOV domain is a three-dimensional structure composed of multiple β-strands and α-helices (Crosson and Moffat, 2001; Moglich et al., 2009). Although LOV1 and LOV2 are structurally similar, they are not functionally identical. LOV1 regulates the light response of LOV2, which mainly controls phosphorylation of the kinase domain (Christie et al., 2015; Okajima, 2016). In the dark, LOV2 non-covalently binds to the FMN chromophore and interacts with STK, inhibiting its catalytic activity. Exposure of blue light alters the helical structures (A′α-helix and Jα-helix) on both sides of LOV2, relieves STK inhibition, eventually leads to phosphorylation of phototropin (Eitoku et al., 2005; Nakasone et al., 2007; Kaiserli et al., 2009) (Fig. 1A). Through high-resolution crystal structure analysis and spectroscopic measurements, blue light was found to induce conformational changes in the LOV2 to enhance STK phosphorylation (Iwata et al., 2003; Halavaty and Moffat, 2007; Liscum et al., 2014).

Phot1 and phot2 are structurally similar, but they are functionally distinct. PHOT1 operates over a wide range of blue light intensities. For example, PHOT1 can mediate light bending of the hypocotyl axis induced by low-fluence blue light (0.01 μmol m^−2^ s^−1^ ~ 1 μmol m^−2^ s^−1^) and high-fluence blue light (> 1 μmol m^−2^ s^−1^), including the first and second positive phototropisms, whereas PHOT2 only mediates second positive phototropism induced by high-fluence blue light (Sakai et al., 2001; Briggs and Christie, 2002). Some Ser and Thr residues in PHOT1 of Arabidopsis have been identified as phosphorylation sites, and high-fluence blue light can induce phosphorylation at these sites, leading to a second positive phototropism. However, no phosphorylation sites associated with the first positive phototropism have been identified under low-fluence blue light (Sakai and Haga, 2012; Christie and Murphy, 2013). Although phot1 and phot2 do not possess a transmembrane domain, they localize to the inner side of the cytoplasmic membrane in the dark (Sakamoto and Briggs, 2002; Kong et al., 2006). Under blue light irradiation, part of the PHOT1 protein detaches from the cell membrane and disperses in the cytoplasm, in contrast, some PHOT2 proteins can be coupled to the Golgi apparatus (Sakamoto and Briggs, 2002; Kong et al., 2006; Wan et al., 2008). Furthermore, phosphorylation site Ser581 of PHOT1 and PHOT2 is ubiquitous in seed plants, ferns, mosses, and green algae (Sullivan et al., 2008). Low-fluence blue light was recently shown to induce the phosphorylation of zmphot1, and five phosphorylation sites were detected (Ser265, Ser291, Ser369, Ser376 and Ser753). Among them, Ser369, Ser291, and Ser376 participate in regulating the first positive phototropism but do not affect the second positive phototropism (Suzuki et al., 2014).

## Plants grow toward the light improve the ability of light capture

The process of plant phototropism, from sensing of blue light to asymmetric growth of the hypocotyl, involves numerous signaling components. Progress has been made on investigating the signaling pathway by which phototropin regulates phototropism. Autophosphorylation of phototropin induced by blue light is considered as the first step in initiating the blue light response in plants (Inoue et al., 2008b). An early study revealed that unilateral blue light induced autophosphorylation of PHOT1 in *oat* (*Avena sativa*) coleoptiles and exhibited a gradient distribution (Salomon et al., 1997). Plants express different phototropin kinase substrates in different tissues to regulate phototropin in response to various physiological responses. Currently, only two substrates have been identified for phototropin kinases: ATP-binding cassette B19 (ABCB19) and PHYTOCHROME KINASE SUBSTRATE 4 (PKS4) involved in regulating hypocotyl phototropism (Christie et al., 2011; Demarsy et al., 2012). Blue light-activated phototropin can phosphorylate ABCB19 and PKS4 to inhibit their activity and enhance phototropism (Christie et al., 2011; Demarsy et al., 2012). Interaction screening revealed that NPH3, RPT2, and PKS1 interact with PHOT1 and participate in the regulation of hypocotyl phototropism (Motchoulski and Liscum, 1999; Harper et al., 2000; Inada et al., 2004). Although NPH3 and RPT2 are involved in PHOT2-mediated phototropism under high-fluence blue light, RPT2 does not interact with PHOT2 in vivo, and the regulatory mechanism remains unclear (Sakai et al., 2000; Lariguet et al., 2006; Zhao et al., 2013; Zhu et al., 2021a). So far, some components of phototropin-mediated phototropism in Arabidopsis have been identified, but the detailed mechanism underlying this process requires further analysis.

### Early signaling events in phototropism

In Arabidopsis, the NPH3/RPT2-like (NRL) and PKS protein families are thought to be involved in phototropin-mediated signal transduction in the early stages of photomorphogenesis. Mutations in *NPH3* causes Arabidopsis lose all-intensity blue light-induced phototropism, indicating that NPH3 regulates both PHOT1-mediated phototropism under low-fluence blue light and the PHOT1- and PHOT2-mediated high-fluence blue light response (Liscum and Briggs, 1996; Motchoulski and Liscum, 1999). The NPH3-encoded protein contains a protein–protein interaction domain broad-complex, tramtrack, bric-a-brac/Pox virus, zinc finger (BTB/POZ), and helix–helix domains at both ends (Motchoulski and Liscum, 1999). In the dark, NPH3 protein localizes to the cytoplasmic membrane and interacts with the *N*-terminus of the phot1 protein through its *C*-terminus (Motchoulski and Liscum, 1999). Under blue light, NPH3 undergoes PHOT1-dependent dephosphorylation, detaches from the plasma membrane, and enters the cytoplasm to form aggregates (Pedmale and Liscum, 2007; Sullivan et al., 2019). In the dark or after prolonged light exposure, NPH3 is phosphorylated and localized again to the plasma membrane (Christie et al., 2018) (Fig. 1B). Recently, two studies explained this process. A conserved *C*-terminal consensus sequence (RxS) of NPH3 is directly phosphorylated by phot1, which promotes phototropism and petiole localization in Arabidopsis. RxS phosphorylation results in changes in the phosphorylation and localization state of NPH3 and triggers binding to 14–3-3. Subsequent binding of the 14–3-3 protein is responsible for light-induced release of NPH3 from the plasma membrane with accompanying NPH3 dephosphorylation (Reuter et al., 2021; Sullivan et al., 2021). The process of NPH3 leaving the plasma membrane is regulated by PHOT1, whereas PHOT2 regulates the stability and repositioning of NPH3 to the plasma membrane to adapt to high-fluence blue light (Zhao et al., 2018). The study showed that under high-fluence blue light, the hypocotyl axis of a *phot1* mutant was bent toward light but the NPH3 protein was not phosphorylated, indicating that PHOT2 does not require phosphorylation of NPH3 to mediate phototropism in Arabidopsis (Tsuchida-Mayama et al., 2008; Zhao et al., 2013). Additionally, in maize, the *NPH3* and *PGP* genes are specifically expressed at the tip of the *maize* coleoptile, similar to the expression pattern observed in Arabidopsis (Matsuda et al., 2011). NPH3 was evaluated to determine its interaction with cullin 3a (CUL3a), a CULLIN3-based E3 ubiquitin ligase complex (CLR3) component. CUL3 is required for blue light-induced ubiquitination of PHOT1, and PHOT1 ubiquitination is thought to be involved in phototropism (Salomon et al., 1997; Thomann et al., 2005; Roberts et al., 2011).

Although phosphorylation of NPH3 is related to phototropism, for plants to achieve maximum response to light, continuous phosphorylation of NPH3 is required, mainly involving RPT2 (Liscum et al., 2014; Haga et al., 2015). RPT2 is homologous to NPH3, and mutation in *RPT2* causes Arabidopsis lose root negative phototropism but still exhibit phototropism under low-fluence blue light (Sakai et al., 2000). RPT2 is localized to the plasma membrane and forms a complex with PHOT1 and NPH3 in vivo (Inada et al., 2004). Low-fluence blue light can induce phototropism in *rpt2* mutants but the phototropism gradually weakens with increasing blue light, indicating that RPT2 promotes phototropism in Arabidopsis under blue light (Sakai et al., 2000; Inada et al., 2004). Furthermore, under prolonged periods of blue light irradiation, RPT2 accumulates, and the phosphorylation and plasma membrane localization of NPH3 are restored in an RPT2-dependent manner (Haga et al., 2015) (Fig. 1B). PHOT1 mediates hypocotyl phototropism under high-intensity blue light depending on RPT2 protein (Sakai et al., 2000; Inada et al., 2004), but RPT2 facilitates the adaptation of PHOT1 to high-intensity blue light remains unclear. RPT2 reportedly binds to the LOV1 domain of PHOT1 and inhibits the phosphorylation and the activity of phot1. RPT2 proteins are stabilized in a phot1-dependent manner under blue light. When phot1 is inactivated, RPT2 are degraded through the ubiquitin–proteasome pathway. In plant, the probable mechanism of RPT2 maintains a moderate activation level in a phot1 dependent manner is to adapt different light intensities (Kimura et al., 2020).

Blue light can induce phosphorylation of phototropins to phosphorylate other proteins. ABCB19 and PKS4 are involved in regulating phototropism as phototropins substrates (Liscum et al., 2014; Okajima, 2016). PKS1, PKS2, and PKS4 belong to the PKS protein family and are important components of the Arabidopsis phytochrome signal transduction pathway (Fankhauser and Chory, 1999; Fankhauser et al., 1999). PKS1, PKS2, and PKS4 regulate the phototropins-mediated blue light response (Lariguet et al., 2006; de Carbonnel et al., 2010). PKS4 localizes to the plasma membrane, interacts with PHOT1 and NPH3 in vivo, and is required for blue light-induced phototropism (Kami et al., 2014). In the dark, PKS4D exists in its dephosphorylated form, blue light induces PHOT1-dependent phosphorylation of PKS4D and negatively regulates phototropism (Demarsy et al., 2012) (Fig. 1B). Single gene mutations in *PKS1*, *PKS2* or *PKS4* have no phenotype, but the hypocotyl curvature is significantly reduced in double mutants, and phototropism is entirely absent in triple mutants, suggesting that these genes have functional redundancy in phototropism (Lariguet et al., 2006). Although the biochemical function of PKS proteins is unclear, these proteins may affect phototropism by regulating auxin signaling (de Carbonnel et al., 2010; Kami et al., 2014).

Root curling in *n*-naphthylphthalamic acid (RCN1) encodes the A1 subunit of Ser/Thr protein phosphatase 2A (PP2A) and interacts with PHOT2 to negatively regulate PHOT2-mediated phototropism in Arabidopsis. However, RCN1 is not involved in PHOT1-mediated phototropism (Tseng and Briggs, 2010). Immunoblot analysis revealed that, when PP2A activity was inhibited, phot2 was dephosphorylated more slowly than in the wild-type background, indicating that phosphorylated PHOT2 is a substrate for PP2A. Although reduced PP2A activity enhanced phot2 activity, it did not enhance dephosphorylation of phot1. Therefore, PP2A may participate in phototropism by regulating the activity of PHOT2 (Tseng and Briggs, 2010).

### Formation of auxin gradient and downstream auxin response

According to the Cholodny-Went theory, the asymmetric distribution of auxin causes plant tropism. When the auxin reporter gene DR5_rev_:GFP was transferred into oat, blue light led to the formation of an auxin concentration gradient in the stem (Salomon et al., 1997; Zhu, 2021b). Many light-bending mutants exhibit defects in auxin-related signaling (Sakai et al., 2012), suggesting that auxin transport is vital for phototropism. The transport and asymmetric distribution of auxin depends on auxin transporters (Kami et al., 2010). Three auxin transporter protein families have been identified in plants, including the auxin efflux carrier PINs family, influx carrier (AUX1) and similar families (LAX), and ABCB auxin transporter (Blakeslee et al., 2005; Whippo and Hangarter, 2006; Robert and Friml, 2009). Eight members of the PIN family of auxin efflux carriers are present in Arabidopsis. PIN1, PIN2, PIN3, PIN4 and PIN7 have long hydrophilic loops and show a polar distribution in the cell membrane; these proteins can transport auxin from the cytoplasm to the extracellular matrix (Zazímalová et al., 2010). Using the auxin reporter gene DR5_rev_:GFP to observe the effect of PINs on the auxin distribution, PIN3 gene mutation was found to weaken auxin accumulation on the dorsal light side of the hypocotyl in Arabidopsis and lead to reduced phototropism (Friml et al., 2002). Phenotypic verification of the multi-mutated material showed that *pin3 pin7* double mutants exhibited weaker phototropism compared to plants with a *pin3* single mutant, indicating that phototropism in the Arabidopsis hypocotyl is mainly regulated by PIN3 and PIN7 (Ding et al., 2011). Although PINs are involved in regulating phototropism, the mechanism by which phototropins regulate PINs is unclear. The protein homologous to phototropin, D6 kinase protein (D6PK), can phosphorylate PINs under well-lit conditions, and auxin transport and tropic responses are partially impaired in phototropic and negative gravitational responses in *D6PKOE* and *d6pk* single and double mutants, suggesting that D6PK is involved in regulating phototropin-mediated phototropism (Willige et al., 2013; Haga et al., 2014). In Arabidopsis, four auxin influx carriers have been identified: AUXIN1(AUX1), LIKE-AUX1(LAX1), LAX2 and LAX3 (Titapiwatanakun and Murphy, 2009; Zazímalová et al., 2010). Mutations in *AUX1* resulted in enhanced phototropism in the roots without affecting phototropism in the hypocotyls (Watahiki et al., 1999). *aux1 lax2 lax3* triple mutant seedlings exhibited reduced phototropism in the hypocotyl axis (Stone et al., 2008). However, there is no direct evidence that phototropins modulate the activity of AUX1/LAX. As described previously, ABCB19, the PHOT1 substrate, can be directly phosphorylated, thereby inhibiting its auxin transport activity. The study showed that exposure to red or blue light for 4 h reduced ABCB19 levels in the hypocotyl, which inhibited auxin transport to the base of hypocotyl (Nagashima et al., 2008a). ABCB19 also interacts with PIN1 to stabilize PIN1 membrane localization and regulate its activity (Blakeslee et al., 2007; Titapiwatanakun et al., 2009). ABCB19 interacts with PINs, and also binds to the auxin efflux carrier inhibitor, *N*-1-naphthyl anthranilic acid (Noh et al., 2001) (Fig. 1B). *N*-1-Naphthyl anthranilic acid can inhibit the phototropism and gravitropism of Arabidopsis hypocotyls and mainly exerts its effect by inhibiting the activity of auxin efflux carrier PINs, which can, in turn, regulate the asymmetric distribution of auxin through ABCB19 (Titapiwatanakun et al., 2009).

However, the mechanism by which phototropins control auxin transport remains unclear. Haga et al. (2014) demonstrated that PINOID kinase (PID) can regulate the phosphorylation state of PINs and affect their localization. In contrast, PID activity is regulated by intracellular Ca^2+^ levels. PHOT1 and PHOT2 can mediate the influx of Ca^2+^ under high- and low-fluence blue light, respectively (Babourina et al., 2002; Zhao et al., 2013). Thus, the regulation of intracellular Ca^2+^ by phototropin may affect auxin transport. Plants can sense auxin through TIR1/AFB and regulate the auxin response to influence phototropism (Mockaitis and Estelle, 2008). Arabidopsis contains six members of the TIR1/auxin-binding F-box (AFB) family of proteins, namely TIR1, AFB1, AFB2, AFB3, AFB4, and AFB5, all of which are localized in the nucleus (Dharmasiri et al., 2005). Quadruple mutants of *tir1 afb1 afb2 afb3* exhibit severe defects in phototropism (Millar et al., 2010). The TIR1V/AFB auxin receptor may respond to the asymmetric distribution of auxin by regulating ARF7 or other transcriptional pathways. Auxins can bring together Aux/IAAs and F-box proteins in the TIR1/AFB family. These F-box proteins are components of the SCF-type E3 ubiquitin protein ligase complex, which transfers activated ubiquitin (Ub) from the E1/E2 enzyme system. Polyubiquitination of Aux/IAAs results in their degradation, which releases the transcription of auxin-related genes (Leyser, 2018). Aux/IAA proteins and co-repressor protein TOPLESS (TPL) can jointly inhibit the transcription of auxin response factor (ARF). NPH4/ARF7 was identified by screening of phototropism-deficient mutants (Harper et al., 2000). The results revealed that AUX/IAA19 is insensitive to auxin-induced degradation. Additionally, ARF7 and AUX/IAA19 can interact with each other, and their interaction states can affect phototropism. ARF7 is an important protein that regulates the auxin response. AUX/IAA19 binds to ARF and inhibits its activity (Tatematsu et al., 2004) (Fig. 1B). Recent studies showed that single mutant of IAA7 and IAA17 also exhibit a phototropism-deficient phenotype, suggesting that auxin signaling is necessary for blue light-induced phototropism (Vandenbussche et al., 2014). Ultimately, the asymmetric distribution of auxin results in asymmetric elongation of cells. In dicotyledonous plants, plasma membrane (PM) H^+^-ATPase can cause acidification of apoplasts. A PH-sensitive α-expansin protein in the apoplastic regulates cell wall ductility (Cosgrove, 2005; Velasquez et al., 2016). KAT1 potassium channels mediate K^+^ reflux to balance acidification of the apoplast in Arabidopsis (Philippar et al., 2004). Additionally, blue light can induce differential expression of ZMK1 (*Zea mays* K^+^ channel 1) in maize sheaths to affect auxin redistribution, suggesting that ZMK1 is involved in regulating phototropism in maize (Fuchs et al., 2003).

### Modulation of phototropism by other photoreceptors

The blue light receptor phototropin mainly mediates the phototropism of angiosperms, and *phot1 phot2* double mutants exhibit slight phototropism after intense blue light irradiation (Sakai et al., 2001; Zhao et al., 2013, 2018, 2020). Mutating both phytochromes and cryptochromes in Arabidopsis resulted in severe phototropism loss (Ohgishi et al., 2004; Tsuchida-Mayama et al., 2010; Zhao et al., 2020), suggesting that, in addition to phototropin, phytochromes and cryptochromes are involved in regulating phototropism in plants. In etiolated Arabidopsis seedlings, phytochromes and cryptochromes mediate red- and blue light-induced *RPT2* expression, respectively, and affect phototropism by regulating *RPT2* expression (Sakai et al., 2000; Tsuchida-Mayama et al., 2010). Blue light can activate PHOT1 and convert PKS4 into phosphorylated PKS4L, and phytochrome can promote dephosphorylation of PKS4L. PKS4L can inhibit phototropism; therefore, phytochrome-promoted dephosphorylation of PKS4L may enhance phototropism (Fig. 1B) (Demarsy et al., 2012). Red light-activated phytochromes can inhibit blue light-induced migration of PHOT1 to the cytoplasm. (Rosler et al., 2007; Han et al., 2008). However, it is not clear how photosensitive pigments are involved in regulating phototropism by affecting the cell membrane localization of PHOT1. To determine the functional specificity in different localizations of phytochrome A, (Kami et al. 2012) constructed a double mutant with an impaired ability to import phytochrome A to the nucleus (*fhy1 fhl*) along with a phytochrome A constitutive nuclear localization protein (phyA-NLS-GFP); they found that *fhy1 fhl* bending toward light was significantly slower than that in the wild type, whereas the bending of the phyA-NLS-GFP hypocotyl toward light was faster than that of the wild-type. These results confirm that phytochrome A is involved in regulating the phototropic growth of plants, which may mainly depend on its localization in the nucleus and transcriptional regulation of related genes.

Phytochromes and cryptochromes can affect phototropism by affecting transcription, as well as by regulating auxin transport. Activation of phytochromes and cryptochromes can significantly inhibit the expression of ABCB19, which inhibits the transport of auxin to the base of Arabidopsis hypocotyls and enhances phototropism of the plants (Blakeslee et al., 2007; Titapiwatanakun et al., 2009). Phytochromes can also regulate other auxin transporters such as PIN1, PIN3, PIN7 and PID (Friml et al., 2002; Devlin et al., 2003; Blakeslee et al., 2007). Although phytochromes affect the expression of most auxin transporters, the mechanism by which phytochromes regulate auxin transporters to affect phototropism has not been explored.

Plants determine their growth direction by integrating phototropism and gravitropism; therefore, factors regulating gravitropism may indirectly influence phototropism (Hangarter, 1997). A single mutation in *phot1* reportedly leads to hypocotyl-negative gravity loss under low-fluence blue light. An analysis of the growth of the *phyA phot1* double mutant reveals that PHYA is necessary for the suppression of gravitropism, as it develops in accordance with gravity. Analysis of the *phot1 cry1 cry2* triple mutant indicated that cryptochrome plays a minor role in this response (Lariguet and Fankhauser, 2004). These results suggest that, at low-fluence blue light, gravitropism is inhibited by the action of phytochromes and to a lesser extent by cryptochromes (Hangarter, 1997; Sakai et al., 2001; Ohgishi et al., 2004; Iino, 2006). Phytochromes and cryptochromes inhibit the negative gravity of hypocotyls, mainly through the transformation of gravity-sensitive endodermal amyloid bodies in hypocotyls into other plastids with chloroplast or white body characteristics after red or far-red light treatment (Kim et al., 2011). However, the mechanism by which plants integrate phototropism and gravitropism to determine growth direction still remains elusive.

## Blue light regulates chloroplast movement to mediate utilization of light signals

In most eukaryotes, organelle mobility and placement are critical factors affecting intracellular dynamics. Plants are sessile but their organelles move rapidly in response to changing environmental conditions and endogenous cues (Suetsugu and Wada, 2007a; Kong and Wada, 2016). One of the plant organelle motions accurately regulated by ambient light conditions is chloroplast movement (Williams et al., 2003). Under fluctuating light environments, chloroplast movement regulates the balance between biomass production and photoprotection (Gotoh et al., 2018). Blue light is the primary light that induces chloroplast movement to optimize light uptake; chloroplasts travel toward low-fluence blue light and aggregate along the oblique perimeter wall (accumulation response), ensuring efficient photosynthesis and biomass production under a wide range of light intensities (Christie, 2007; Suetsugu and Wada, 2007a, 2013; Gotoh et al., 2018). In addition, chloroplasts build up along the anticlinal wall, decreasing the incidence of high-fluence blue light damage (avoidance response) (Kasahara et al., 2002; Sztatelman et al., 2010; Davis and Hangarter, 2012; Cazzaniga et al., 2013; Kong and Wada, 2016). In the dark, chloroplasts spread to the bottom of the cell, although the physiological importance of this distribution is unknown.

### Mechanism of chloroplast movement to enhance light-capturing ability of plant

Arabidopsis contains two blue light photoreceptors, phot1 and phot2, mediate chloroplast accumulation responses and regulate by two phototropin-interacting proteins, NCH1 and RPT2 (Suetsugu et al., 2016b; Wang et al., 2021; Suetsugu et al., 2016b). The accessory protein, auxin-like 6 or j-domain protein required for chloroplast accumulation response 1 (JAC1), is necessary for the chloroplast accumulation response. *jac1* mutants were defective in the response to chloroplast accumulation, similar to *rpt2 nch1* plants (Suetsugu et al., 2005, 2016b). Weak chloroplast motility 1 (WEB1) and impaired plastid motility 2 (PMI2) are two interacting coiled-coil proteins. *web1* and *pmi2* mutants exhibited attenuated chloroplast avoidance responses under blue light (Luesse et al., 2006; Kodama et al., 2010). Furthermore, WEB1/PMI2 inhibited accumulation responses by inhibiting JAC1 activity under high light (Suetsugu and Wada, 2017). PMI1, a C2 domain protein, is essential for chloroplast movement, and the *pmi1* mutant exhibits substantially impaired chloroplast movement (DeBlasio et al., 2005; Suetsugu et al., 2015).

**Fig. 2:**
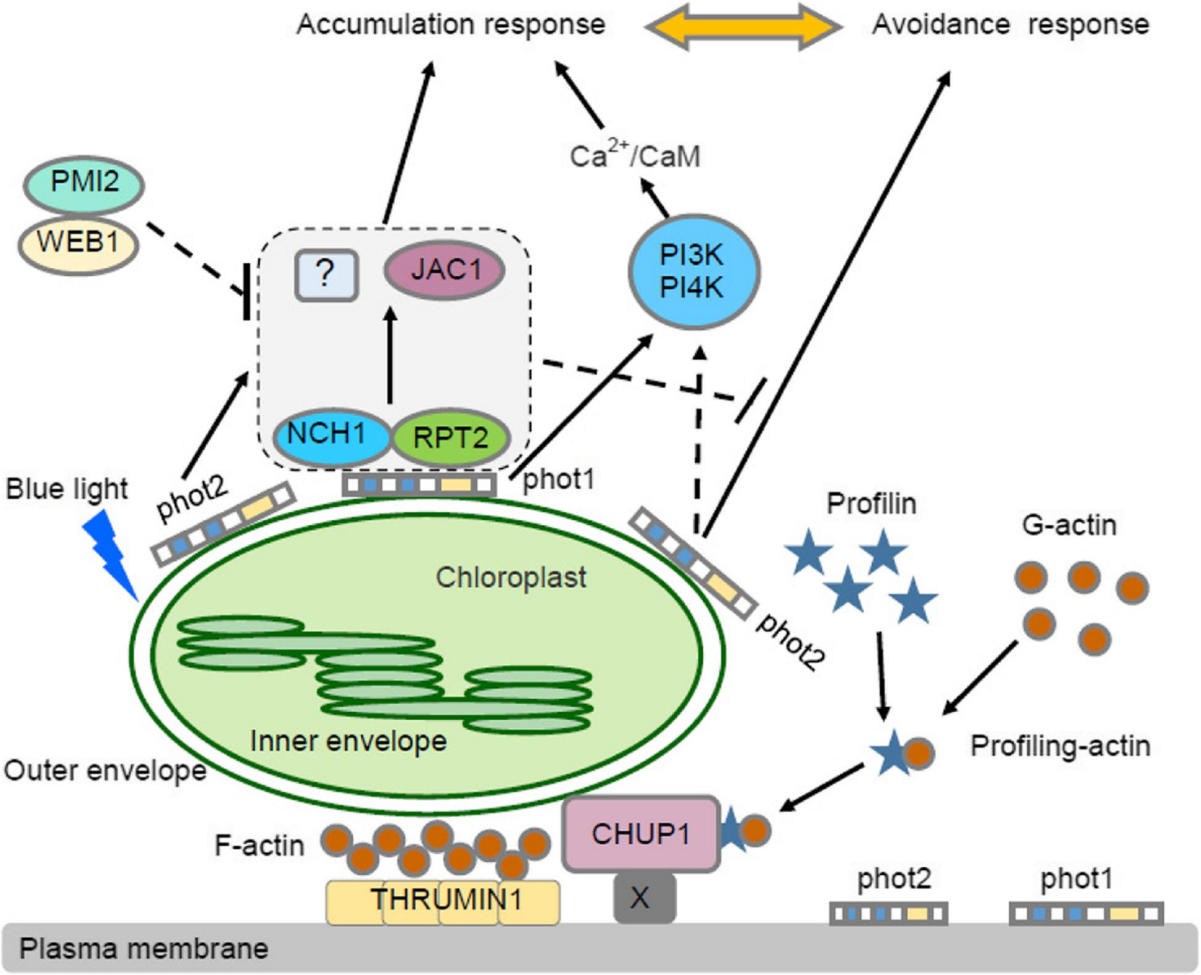
Model of chloroplast accumulation and avoidance responsive molecular processes mediated by phototropin pathway and cp-actin regulation. Under low fluence blue light circumstances, photoreceptors PHOT1 and PHOT2 mediate accumulation responses via RPT2, NCH1/PI3K activation. RPT2 and NCH1 may control JAC1-dependent and -independent pathways, whereas PI3K controls the Ca^2+^/CaM route. Under high fluence blue light conditions, the WEB1/PMI2 complex suppresses the accumulation response controlled by RPT2/NCH1 and JAC1, causing avoidance responses directly mediated by PHOT2 as well as PI4K activation of the Ca^2+^/CaM pathway. Signals for the accumulation and avoidance responses are generated by photoreceptors and received by a signal receptor that is most likely connected with CHUP1, which binds the chloroplast to the plasma membrane via an unknown membrane protein (labeled X). The inclusion of profilin-bound actin initiates a possible CHUP1-dependent polymerization of cp-actin filaments. KAC works with CHUP1 to increase cp-actin polymerization or to maintain cp-actin filaments, at least in the accumulation responses. THRUMIN1, which localizes to the plasma membrane, bundles polymerized cp-actin filaments, resulting in cp-actin filaments that are fixed to the plasma membrane. As a result, as long as cp-actin is polymerized, the chloroplast will migrate forward

Phosphoinositide (Pi) signaling in plants regulates developmental processes and stress responses by influencing the actin structure and vesicle trafficking (Xue et al., 2009). When exposed to blue light, neomycin and U73122 inhibit the phosphatidylinositol 4,5-bisphosphate [PI (4,5) P2]-PLC pathway and PHOT2-mediated chloroplast accumulation and avoidance responses. Inactivation of PI3K and PI4K with wortmannin and LY294002 severely affected the cumulative responses activated by weak blue light irradiation, but had little effect on avoidance responses activated by strong blue light. This result suggests that the PI (4,5) P2-PLC pathway is involved in light-avoidance movement of PHOT2 signaling and that PI3K and PI4K are required for the accumulation response induced by PHOT1 and PHOT2. These phosphoinositides regulate cytosolic Ca^2+^ signaling during chloroplast movement (Aggarwal et al., 2013a, b) (Fig. 2) and ultimately regulate the chloroplast light-gathering movement through chloroplast actin filaments.

### Chloroplast movement system based on CP-actin production

Chloroplasts use two separate cytoskeletal actin filaments and microtubules for dynamic movement and placement, similar to other key subcellular organelles such as the nucleus, mitochondria, endoplasmic reticulum and Golgi apparatus (Sato et al., 2001; Takagi, 2003; Kong and Wada, 2011). Chloroplast actin filaments (CP-actin) are the most important factors involved in chloroplast movement. Although the mechanism by which chloroplast movement generates force is unclear, studies have suggested that CP-actin polymerization generates force (Kadota et al., 2009) (Fig. 2). Since the discovery of CP-actin filaments, the understanding of the mechanism of chloroplast movement has substantially improved. CP-actin filaments are found specifically between the plasma membrane and chloroplasts. Asymmetric distributions of CP-actin filaments around the chloroplast periphery are quickly established, particularly during avoidance movement, by rapidly depolymerizing CP-actin filaments in the opposite direction of the moving chloroplast in the posterior region but strongly in the anterior region. CP-actin filament dynamics involves rapid severing the filaments to shorter lengths and motility of the fragmented CP-actin filaments, which is required to generate their asymmetric distribution. PHOT2 is a critical and the primary photoreceptor involved in regulating CP-actin filament dynamics, whereas PHOT1 plays a minor role. In Arabidopsis, actin filaments are primarily used for chloroplast transport (Takagi, 2003). Important genes involved in chloroplast light relocation have been identified, some of which include chloroplast abnormal localization 1 (CHUP1), kinesin-like protein for actin-based chloroplast movement (KAC), and glutaredoxin family protein (THRUMIN1)*.* These investigations have considerably expanded the understanding of the regulatory roles of various protein components in the polymerization and/or maintenance of CP-actin and its force-generating mechanisms (Kong and Wada, 2011, 2014; Suetsugu and Wada, 2016).

Analysis of the *chup1* mutant in Arabidopsis revealed the key role of the CHUP1 protein in removing chloroplasts from the plasma membrane and depositing them at the bottom of the cell without moving under any light conditions (Oikawa et al., 2003; Wada and Suetsugu, 2004; Higa et al., 2014). The hydrophobic section of CHUP1 protein binds to the chloroplast outer membrane, which can connect the chloroplast to the coiled-coil region of the plasma membrane, F-actin binding site, and *C*-terminal conserved region (Oikawa et al., 2008; Higa et al., 2014). Importantly, CHUP1 binds to contouring (Schmidt von Braun and Schleiff, 2008), which is a tiny actin-binding protein that promotes actin assembly at the barbed end (Kong et al., 2013). Furthermore, CHUP1 is solely involved in actin-mediated motility but not in microtubule-mediated motility (Usami et al., 2012).

In general, typical kinesins are plus-end oriented kinesins with an *N*-terminal motor domain. *KAC* encodes a kinesin-like microtubule motor protein. KAC proteins are members of the kinesin-14 family and contain a minus-end oriented kinesin with a *C*-terminal motor domain. CP-actin-dependent chloroplast mobility is required, although the specific chemical mechanism is unknown (Suetsugu et al., 2010). The Arabidopsis genome contains two *KAC* genes: *KAC1* and *KAC2*. The *kac1 kac2* double mutant plant did not contain CP-actin filaments, and chloroplasts were shed from the plasma membrane (Shen et al., 2015). Notably, CP-actin filaments were absent from chloroplasts in *kac1 kac2* double mutant leaves. There was also a considerable avoidance reaction, although no cumulative response was observed (Suetsugu et al., 2016a). The mechanisms governing chloroplast motor avoidance responses in *kac1 kac2* double mutant plants remain unclear. Although CP-actin filaments are necessary for chloroplast light displacement, the blue light-induced avoidance responses in *kac1 kac2* double mutants are controlled by an unknown actin-dependent mechanism (Higa et al., 2014).

THRUMIN1 is an actin-bundling factor that is regulated by light and involved in chloroplast mobility. This protein contains an inherently disordered area at its *N*-terminus. The *C*-terminus contains a glutaredoxin-like and probable zinc-binding cysteine-rich domain. Through its myristoylated *N*-terminus, THRUMIN1 localizes to the plasma membrane and alters actin filaments in a blue light- and phototropin-dependent manner (Whippo et al., 2011). Importantly, THRUMIN1 colocalized with CP-actin filaments in avoidance reactions. Accordingly, *thrumin1* mutant cells exhibited defects in CP-actin filament reorganization during avoidance responses (Kong et al., 2013).

### Role of reactive oxygen species in chloroplast avoidance response

**Fig. 3:**
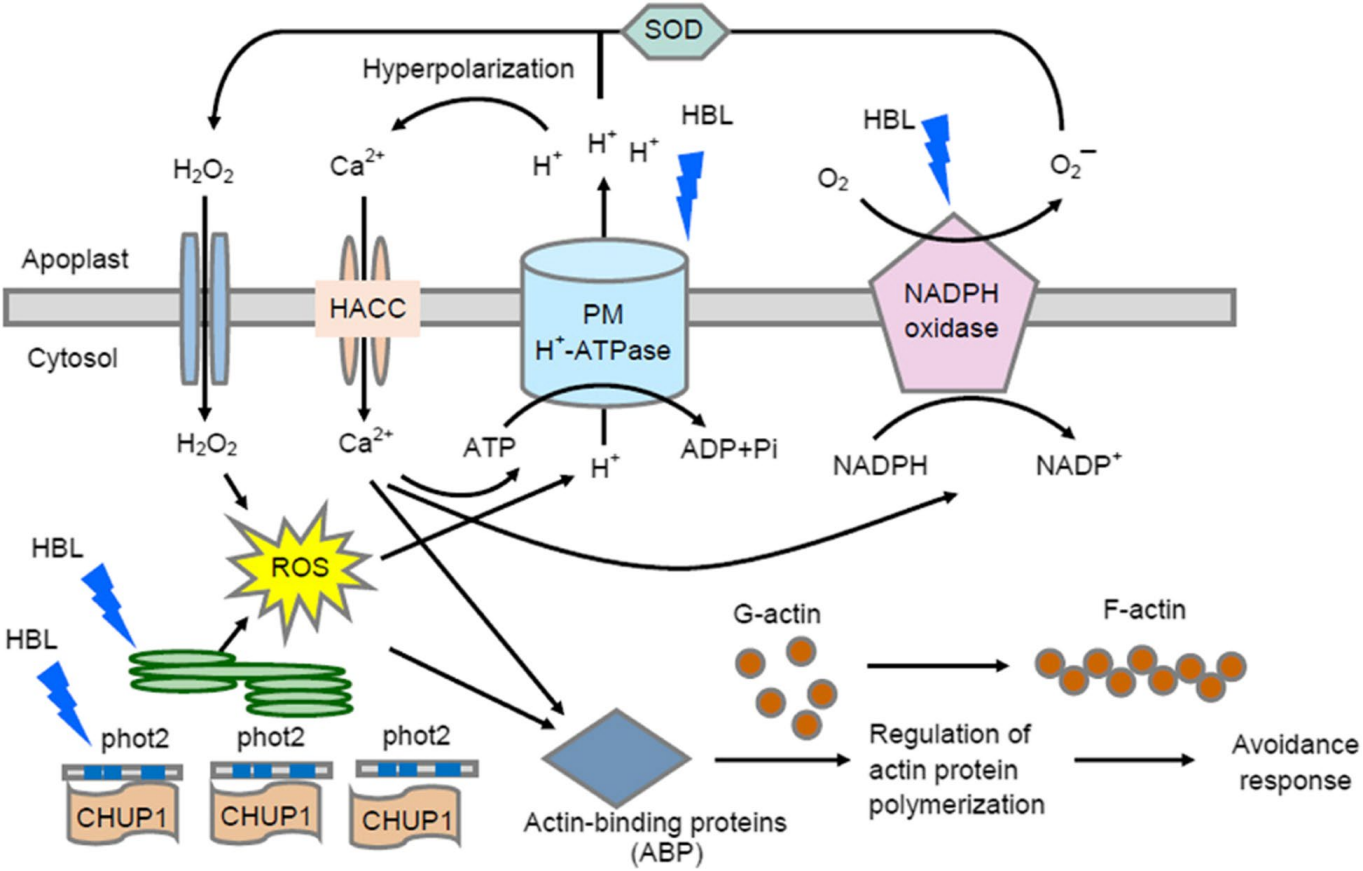
ROS-dependent signaling cascade involved in chloroplast avoidance movement induced by HBL. High blue light (HBL) exposure causes chloroplast thylakoid to produce several kinds of ROS as a result of electron (e) spillover at the photosynthetic electron transport chain (ETC). Chloroplast thylakoid-derived ROS accumulate in the cytosol after being converted to H_2_O_2_ and diffusing through chloroplast membranes. HBL, on the other hand, activates both NADPH oxidase and PM H^+^-ATPase, increasing the rate of O_2_^−^ generation and H^+^ transport across the plasma membrane. Aside from spontaneous processes, NADPH oxidase-generated O_2_^−^ is transformed to H_2_O_2_ by SOD, which uses the H^+^ accessible in the apoplast region due to PM H^+^-ATPase activity. De novo generated H_2_O_2_ diffuses through the plasma membrane, forming a cytosolic ROS pool alongside chloroplast-derived ROS. The accumulating ROS activates Ca^2+^-channels, allowing Ca^2+^ to enter the plasma membrane. Furthermore, the membrane hyperpolarization caused by enhanced PM H^+^-ATPase activity promotes HACCs (hyperpolarization-activated Ca^2+^ channels) and allows Ca^2+^ enter the cytosol. As a result, a threshold Ca^2+^_[cyt]_ is formed, which contains Ca^2+^ released from endosomes. Ca^2+^_[cyt]_ modulates the activity of NADPH oxidase and PM H^+^-ATPase by binding to EF-hand motifs and influencing the phosphorylation of various amino acids, resulting in a positive feedback loop. The HBL-induced buildup of ROS inside the cytosol and the threshold Ca^2+^_[cyt]_ then influences actin polymerization either directly by changing actin amino acids or indirectly by influencing the activities of actin-binding proteins (ABPs). Because of the changed polymerization/depolymerization, chloroplasts can migrate along the plasma membrane to limit excessive light absorption

Reactive oxygen species (ROS) have long been thought to be toxic to plants and mammals by causing lipid peroxidation, DNA damage, and aging (Popa-Wagner et al., 2013; Mittler, 2017). However, ROS can also participate in signaling pathways such as those involved in plant growth and development (Mittler, 2017; Qi et al., 2017). ROS can drive changes in redox reactions, such by increasing the levels of antioxidants and ascorbic acid (Asc) or reducing glutathione (GSH) in chloroplasts (Heyneke et al., 2013), which may be key factors to protecting the body from high-intensity light. However, most of the roles of ROS have not been investigated in detail (Szymańska et al., 2017). ROS function in actin filament polymerization/stabilization in plant systems in neural growth cones, where ROS reduce the content, kinetics, and contractility of F-actin (Munnamalai et al., 2014; Wilson and González-Billault, 2015). At normal physiological concentrations, ROS likely act as signals for chloroplast movements in dark. High blue light can cause ROS (produced by high-energy concentrations to induce NADPH oxidase) to regulate chloroplast light-avoiding movement by regulating the polymerization or depolymerization of actin filaments (Munnamalai et al., 2014; Wilson and González-Billault, 2015). The accumulating ROS also activates Ca^2+^-channels, allowing Ca^2+^ to enter the cytosol, and increase in cytosolic free Ca^2+^ concentration ([Ca^2+^]_cyt_) (Pei et al., 2000). A higher[Ca^2+^]_cyt_ likely to have a feedback regulation on the activity of NADPH oxidase and also influences actin polymerization either directly by changing actin amino acids or indirectly by influencing the activities of actin-binding proteins (ABPs), which can result in actin polymerization or depolymerization (Hepler, 2016; Majumdar and Kar, 2018, 2020). A plausible model has been suggested and additional experiments is necessary for verification (Fig. 3).

## Photosynthetic states are regulated by phototropins-mediated plant movement

Phototropins are commonly observed in higher and lower plants, in addition to mediate phototropism and chloroplast movement, phototropins mediate stomatal opening, leaf extension, and leaf positioning in Arabidopsis (Kagawa and Wada, 2000; Elliott et al., 2004; Christie, 2007). Light-induced chloroplast movement is among the most important responses for utilizing photosynthetic light (Suetsugu and Wada, 2012). The regulation of chloroplast movement by phototropin under weak light conditions can promote light capture. PHOT1 is more sensitive than PHOT2 in regulating chloroplast movement, as PHOT2 requires a higher light intensity for activation (Wada, 2013). In Arabidopsis, the accumulation response is regulated by PHOT1 and PHOT2 (Sakai et al., 2001), whereas the avoidance response is regulated mainly by PHOT2 (Jarillo et al., 2001; Kagawa et al., 2001). Blue light can induce accumulation of RPT2, which not only stabilizes PHOT1 to regulate phototropism (Kimura et al., 2020), but can also be regulated by PHOT1 and PHOT2 to mediate chloroplast accumulation (Jarillo et al., 2001; Kagawa et al., 2001). RPT2 not only regulates phototropism but also mediates chloroplast movement. Therefore, RPT2 may play a key role in cross-regulation of phototropism and chloroplast movement. Phototropins can mediate the opening of plant stomata under blue light and regulate CO_2_ absorption and water loss by integrating photosynthesis and transpiration under different light intensities. Stomatal opening is regulated by the functional redundancy of PHOT1 and PHOT2 (Kinoshita et al., 2001; Takemiya et al., 2013). Leaf extension is also a response that improves light capture under low light conditions. The leaf of the *phot1* and *phot2* mutants expanded more than those of the double mutants at a high fluence light (Ohgishi et al., 2004). The *phot1 phot2* mutant is known to have small curly leaves when grown under white light (Kinoshita et al., 2001; Sakai et al., 2001; Sakamoto and Briggs, 2002). These results suggest that phot1 and phot2 regulate the photomorphogenic response of leaf tissue. In addition, phototropins can adjust Arabidopsis leaf positioning in response to light signals, such that plants can maintain a stable state to obtain light (Inoue et al., 2005). The importance of phototropin-mediated responses in photosynthetic performance and plant growth has been examined using mutant Arabidopsis plants defective in phototropin-mediated responses. Under weak light conditions, *phot1* mutant exhibit reduced photosynthetic performance and growth by reducing chloroplast accumulation, weakening stomatal opening, and causing leaf curling (Takemiya et al., 2005). The *nph3* mutant is also impaired in photosynthesis and plant growth under weak BL conditions. This mutant is also defective in leaf extension (Inoue et al., 2008a). In phototropin-mediated reactions, NPH3 and RPT2 are not only involved in regulating phototropism, but also promote photosynthesis by influencing other reactions. However, it is unclear whether the signals of different light reactions and crosstalk. We evaluated the phototropin-mediated light response and found that plants respond to natural light fluctuations by adjusting their photosynthetic state for growth. Phototropins can improve light harvesting and reduce light damage to plants by regulating microscopic movements, such as stomatal opening and chloroplast movement, as well as through macroscopic regulation of phototropism and leaf positioning, enhancing plant stress resistance.

## Concluding remarks and future perspectives

In this review, we discussed the structure and physiological function of blue light receptor phototropins, and the downstream signal transmission is involved in phototropism, chloroplast accumulation and avoidance responses. Although the roles of photoreceptors and important signaling components such as NPH3, RPT2, and PKSs in plant phototropism are becoming increasingly clear, the molecular relationship between photoreceptor activation and auxin transport or signaling require further analysis. Furthermore, chloroplast orientation and fast movement may involve blue light-mediated formation of the asymmetric distribution of CP-actin filaments in the chloroplast and many components involved in regulating CP-actin filament dynamics have been revealed. However, the molecular activities of these protein components, such as the communication from photoreceptors to chloroplasts and the process of power production, remain unclear. Finally, to determine the physiological mechanism of phototropic growth in nature and chloroplast movement under various light conditions (for plants growing under strong, weak, or fluctuating light conditions), focus on crops how to shield them from high-intensity light and enhance the optimum use of dim light, which will provide a theoretical foundation for enhancing the ability of crops to increase light harvest and stress tolerance.

## Data Availability

Data sharing is not applicable to this article as no new data were created or analyzed in this study.

## Abbreviations

ABPs: Actin-binding proteins
BL: Blue light
CAM: Calmodulin
Cp-actin filaments: Chloroplast-actin filaments
Cry: Cryptochrome
ETC: Electron transport chain
FMN: Flavin mononucleotide
HACC: Hyperpolarization-activated Ca^2+^ channels
LOV: Light, oxygen or voltage loca
PHOT: Phototropin
PM: Plasma membrane
PtdIns3K、PtdIns4K: Phosphatidylinositol kinases
ROS: Reactive oxygen species
TIRFM: Total internal reflection fluorescence microscope
